# Modeling of the Atomic Diffusion Coefficient in Nanostructured Materials

**DOI:** 10.3390/e20040252

**Published:** 2018-04-05

**Authors:** Zhiqing Hu, Zhuo Li, Kai Tang, Zi Wen, Yongfu Zhu

**Affiliations:** 1 Roll Forging Research Institute, Jilin University, Changchun 130022, China; 2 School of Materials Science and Engineering, Jilin University, Changchun 130022, China; 3 Key Laboratory of Automobile Materials, Ministry of Education, Jilin University, Changchun 130022, China

**Keywords:** nanostructured materials, diffusion coefficient, grain boundary energy

## Abstract

A formula has been established, which is based on the size-dependence of a metal’s melting point, to elucidate the atomic diffusion coefficient of nanostructured materials by considering the role of grain-boundary energy. When grain size is decreased, a decrease in the atomic diffusion activation energy and an increase in the corresponding diffusion coefficient can be observed. Interestingly, variations in the atomic diffusion activation energy of nanostructured materials are small relative to nanoparticles, depending on the size of the grain boundary energy. Our theoretical prediction is in accord with the computer simulation and experimental results of the metals described.

## 1. Introduction

In the field of materials science, much attention has been given to nanoparticles (NPs) and nanostructured materials (NSs) because of their distinct chemical, physical, and mechanical properties [1,2]. NSs are polycrystalline materials consisting of grains smaller than 100 nm in size, while NPs refer to ultrafine free particles. NPs possess free surfaces, while the interfaces of NSs consist of grain boundaries between grains. When *L* (*L* denotes the diameter of NPs, or the grain size of NSs) is less than the critical value (~10–20 nm), 50% of the atoms are at the free surfaces or grain boundaries [3]. Evidence shows that the thermodynamic properties of NSs and NPs become size-dependent when *L* enters the nanometer scale [4,5,6], resulting in different thermodynamic performances relative to their bulk counterparts. One important reason for this is that the atomic coordination imperfection exists at the surfaces or interfaces of the NSs/NPs. Since the coordination number of the atoms on the grain boundaries is larger than the free surfaces, the grain boundary energy (*γ*_gb_) is less than the surface energy (*γ*_sv_) [7]. NSs are widely used, but receive more attention in magnetic data storage, ultra-large-scale integration, thermoelectric power generation, and structural engineering [8,9,10]. In order to provide the critical information for the above applications, the atomic diffusion property of NSs should be taken into consideration regarding diffusion processing [2,11].

In materials engineering, atomic diffusion, either by the migration or jump of random individual atoms, is an important topic in solid-state physics and chemistry, materials science, and physical metallurgy [12]. For the purposes of materials development and engineering, it is necessary to gain a complete understanding of diffusion in different materials. As an example, Guisbiers and Buchaillot [13] claimed that thermo-mechanical behavior, such as creep, is an important factor affecting device reliability at the nanoscale. Different stages of creep are attributed to grain boundary diffusion, dislocation movement, and lattice diffusion. According to the so-called diffusion spectrum of metals [12], self-diffusion in a regular lattice is several orders of magnitude slower than diffusion in dislocations and grain boundaries. Moreover, the diffusion shortcuts showed low diffusion activation enthalpy, and the diffusivity discrepancy increased as the temperature decreased. Measurement results show that the diffusion coefficient *D_T_* (*L*,*T*) of NSs increased as *L* declined [11,14], where *T* denotes the temperature. The *D_T_* (*L*,*T*) of nanostructured Cu is about 10~16 orders of magnitude higher than the bulk Cu when *L* is approximately 13 nm [15], while an increase in *D_T_* (*L*,*T*) from 0.33 × 10^−8^ m^2^/s to 1.0 × 10^−8^ m^2^/s was observed for nanostructured Ni when *L* decreased from 9 nm to 4 nm [16]. Owing to this, NSs are usually used in diffusion processing, including coating [16,17], nitriding [18], and carburizing [19], significantly improving the processing efficiency [20]. Since NSs consist of nanoscaled grains with a solid–solid interface or grain boundary between them [21], the increase in the diffusion shortcuts should have had a significant impact on raising the diffusivity. Considering that the coordination imperfection exists at the interface, the diffusion might also have been influenced. Compared to NPs with free surfaces, the coordination imperfection should have been weak at the interface, influencing the diffusion of NSs. However, a theoretical way to elucidate this has still remained unavailable.

As reviewed by Laughlin and Hono [12], different diffusion coefficient types were found in the experimental observation, such as random walk, tracer self-diffusion, trace impurity diffusion, intrinsic diffusion, and interdiffusion. In any case, the diffusion coefficients can be described by the Arrhenius diffusion formula [22]. Therefore, the diffusion coefficient of atoms in bulk materials *D_T_* (*∞*,*T*) can be expressed as *D_T_* (∞,*T*) = $D_{T}^{0}\left(\infty \right)\exp\left[-E_{a}\left(\infty \right)/\left(RT\right)\right]$, where ∞ denotes the bulk size, $D_{T}^{0}$ is the pre-exponential constant, the activation energy is denoted by *E_a_*, and the ideal gas constant is denoted by *R*. Upon the atomic diffusion in NPs, the surface effect on the diffusion coefficient of atoms, denoted as $D_{T}^{\mathrm{NP}}\left(L,T\right)$, has been considered and modeled [14]. According to this work, extending the above *D_T_* (∞,*T*) expression into the nanometer regime, $D_{T}^{\mathrm{NP}}\left(L,T\right)$ can be given as [22],

(1) $$D_{T}^{\mathrm{NP}}\left(L,T\right)=D_{T}^{0}\left(L\right)\exp\left[-E_{a}^{\mathrm{NP}}\left(L\right)/\left(RT\right)\right]$$

where $E_{a}^{\mathrm{NP}}\left(L\right)=E_{a}\left(\infty \right)T_{m}^{\mathrm{NP}}(L)/T_{m}(\infty )$ with *T_m_* being the melting point. Moreover, based on Lindemann’s criterion and Mott’s equation of vibrational entropy, the size-dependent *T_m_* of NPs denoted as $T_{m}^{\mathrm{NP}}(L)$ is formulated as [23]

(2) $$T_{m}^{\mathrm{NP}}(L)=T_{m}(\infty )\exp[-(\alpha_{\mathrm{NP}}-1)/(L/L_{0}-1)]$$

where *α*_NP_ = 2*S*_vib_(∞)/3*R* + 1 denotes the surface effect factor, *S*_vib_ is the vibrational contribution of the overall melting entropy of the bulk crystals. *L*_0_ is the critical diameter of a nanoparticle, where almost all atoms are located on the surface, where *L*_0_ = 2(3 − *d*)*h*, *d* represents the dimension of the nanoparticle, with *d* = 0 for nanoparticles, *d* = 1 for nanowires, *d* = 2 for thin films, and *h* is the atomic diameter [24]. In light of this, $T_{m}^{\mathrm{NP}}(L)$ decreased as *L* was lowered [23]. An important reason for this change is relevant to the decrease in atomic cohesive energy associated with the atomic coordination imperfection at the surfaces. Based on Equation (1), and with the help of Equation (2), as *L* decreases, the diffusion coefficient $D_{T}^{\mathrm{NP}}\left(L,T\right)$ is increased by several orders of magnitude [14,25]. When *L* is as small as several nanometers, $D_{T}^{\mathrm{NP}}\left(L,T\right)/D_{T}\left(\infty ,T\right)$ could be greater than 10^10^ due to the drop of $E_{a}^{\mathrm{NP}}(L)$. Moreover, the diffusion temperature could be lowered by several hundred degrees when a constant diffusion coefficient is required [14]. This increase is believed to be attributed to the surface energy, which is associated with the coordination imperfection at the surface.

However, Equation (1) is not able to depict the diffusion in NSs, because the grain boundary energy is lower than the surface energy. Therefore, in order to explain the change in the experimental data of the diffusion coefficient of NSs, it is necessary to establish the diffusion model $D_{T}^{\mathrm{NS}}\left(L,T\right)$ by considering the role of grain boundary energy.

In this paper, a formula will be proposed to elucidate the size-dependence of the diffusion coefficient in NSs in reference to NPs. Based on the modeling of the atomic diffusion activation energy of NSs, $E_{a}^{\mathrm{NS}}\left(L\right)$, the corresponding diffusion coefficient $D_{T}^{\mathrm{NS}}\left(L,T\right)$ was worked out with the help of $T_{m}^{\mathrm{NS}}(L)$. The role played by the grain boundary energy will be considered. The model predictions show good agreement with the experimental results.

## 2. Model

Although the interfaces of the NSs (grain boundaries) and the NPs (free surfaces) are different, grains in the NSs have a similar crystalline structure to the NPs. With reference to NPs, the self-diffusion or intrinsic diffusion coefficient $D_{T}^{\mathrm{NS}}\left(L,T\right)$ of NSs can thus be expressed by extending the $D_{T}^{\mathrm{NP}}\left(L,T\right)$ expression to the NSs case, and it reads [22]

(3) $$D_{T}^{\mathrm{NS}}\left(L,T\right)=D_{T}^{0}\left(L\right)\exp\left[-E_{a}^{\mathrm{NS}}\left(L\right)/\left(RT\right)\right]$$

On the basis of Equation (3), $D_{T}^{\mathrm{NS}}[L,T_{m}^{\mathrm{NS}}(L)]$ = *D_T_* [∞, *T_m_*(∞)] is assumed [22], and one sees $D_{T}^{\mathrm{NS}}[L,T_{m}^{\mathrm{NS}}(L)]$ = $D_{T}^{0}(L)\exp\{-E_{a}^{\mathrm{NS}}(L)/[RT_{m}^{\mathrm{NS}}(L)]\}$ = $D_{T}^{0}(\infty )\exp\{-E_{a}(\infty )/[RT_{m}(\infty )]\}$. In terms of the point defect mechanism [14], $E_{a}^{\mathrm{NS}}(L)$ in this expression means the activation enthalpy with Δ*H* = $E_{a}^{\mathrm{NS}}(L)$, and $D_{T}^{0}$ is an amount proportional to *λ*^2^, *Z* and exp(Δ*S*_NS_/*R*), where *λ* is the interplanar crystal spacing, *Z* is the nearest neighbor gap number and Δ*S*_NS_ is the activation entropy. The sizes of *λ*, *Z*, and Δ*S*_NS_ do not affect the thermal vibration energy. According to the thermodynamic knowledge [14], *T* [∂Δ*S*_NS_(*L*)/∂*T*]*_P_* = ${\left[\partial E_{a}^{\mathrm{NS}}(L)/\partial T\right]}_{P}$, where *P* = 4*f*/*L* is the internal pressure of the sphere particles under a specific size. Hence, Δ*S*_NS_(*L*) changes with $E_{a}^{\mathrm{NS}}(L)$. However, regarding the activation process, the change in Δ*S*_NS_ (*L*) caused by the change of vibrational frequency is less than 5%, which is quite small even when *L* is changed from the bulk size to 4–6 nm [26]. $E_{a}^{\mathrm{NS}}(L)$ is therefore temperature independent. $D_{T}^{0}(L)$ is a weak function of *L*, but the exponential effect $\exp\left\{-E_{a}^{\mathrm{NS}}\left(L\right)/\left[RT_{m}^{\mathrm{NS}}\left(L\right)\right]\right\}$ on $D_{T}^{0}(L)$ is very strong. As a first order approximation, assuming $D_{T}^{0}(L)\approx \;D_{T}^{0}(\infty )$ [14], we thus have,

(4) $$E_{a}^{\mathrm{NS}}\left(L\right)=E_{a}\left(\infty \right)T_{m}^{\mathrm{NS}}(L)/Tm\;(\infty )$$

In light of Equation (4), $E_{a}^{\mathrm{NS}}(L)$ can be worked out if $T_{m}^{\mathrm{NS}}(L)$ is known. In fact, the $T_{m}^{\mathrm{NS}}(L)$ function can be deduced based on the expression of $T_{m}^{\mathrm{NP}}(L)$ in Equation (2), by considering the grain boundary energy effect. According to our previous work [11], it reads,

(5) $$T_{m}^{\mathrm{NS}}(L)=T_{m}(\infty )\exp[-\delta (\alpha_{\mathrm{NP}}-1)/(L/L_{0}-1)]$$

with *δ* = 1/{1 + [*γ*_sv_(∞)/*γ*_gb_(∞) − 1]*α*_NP_} where *δ* is an additional parameter showing the role of grain boundaries relative to free surfaces. Thus, by substituting Equation (5) into Equation (3) with the help of Equation (4), we get,

(6) $$D_{T}^{\mathrm{NS}}(L,T)=D_{T}^{0}(L)\exp[-\frac{E_{a}(\infty )}{RT}\exp[-\delta (\alpha_{\mathrm{NP}}-1)\times \frac{1}{L/L0-1}]]$$

In light of Equation (6), $D_{T}^{\mathrm{NS}}(L,T)$ can be worked out as the function of *L*.

## 3. Results and Discussion

**Figure 1:**
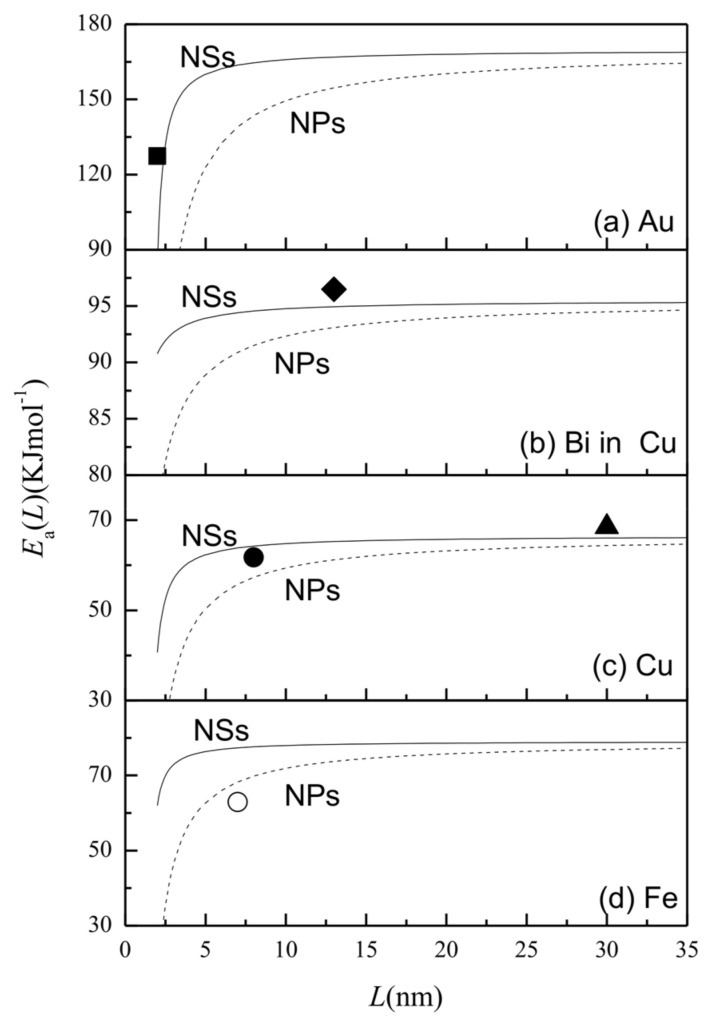
$E_{a}^{\mathrm{NS}}(L)$ as the function of *L* (solid) in terms of Equations (4) and (5) for (**a**) Au, (**b**) Bi in Cu, (**c**) Cu, and (**d**) Fe, where Bi in Cu in (**b**) means the diffusion of Bi in nanostructured Cu and Au in (**a**), Cu in (**c**), and Fe in (**d**) give the self-diffusion data. $E_{a}^{\mathrm{NP}}\left(L\right)$ functions (dashed) are also plotted with Jiang’s prediction [14] for comparison. The symbols show experimental results: (**a**) ■ [22] for Au nanostructured materials (NSs), (**b**) ♦ [15] for Bi in Cu NSs, (**c**) ● [27] and ▲ [28] for Cu nanoparticles (NSs), and (**d**) ○ [4] for Fe NPs. The parameters for the calculation are shown in Table 1.

Figure 1 shows the $E_{a}^{\mathrm{NS}}(L)$ functions of (a) Au, (b) Bi in Cu, (c) Cu, and (d) Fe in terms of Equations (4) and (5) in comparison with experimental results, where Au in (a), Cu in (c), and Fe in (d) give the self-diffusion data for the NSs, where Bi in Cu in (b) means the diffusion of Bi in nanostructured Cu. $E_{a}^{\mathrm{NP}}(L)$ is also plotted in terms of Jiang’s prediction [14] for comparison. $E_{a}^{\mathrm{NS}}(L)$ and $E_{a}^{\mathrm{NP}}(L)$ decreased when *L* was lowered, with $E_{a}^{\mathrm{NP}}(L)$ < $E_{a}^{\mathrm{NS}}(L)$ < *E_a_* (∞). A significant decrease in $E_{a}^{\mathrm{NS}}(L)$ occurred at about *L* ≈ 5 nm, although the decrease of $E_{a}^{\mathrm{NP}}(L)$ was observed at around *L* ≈ 10 nm. When *L* > 10 nm for NSs or *L* > 20 nm for NPs, *E_a_* (*L*) → *E_a_* (∞). Compared to the bulk case, the decrease in $E_{a}(L)$ should be relevant to large thermal vibrational energies of atoms at the surfaces or grain boundaries. The observation that $E_{a}^{\mathrm{NS}}(L)$ > $E_{a}^{\mathrm{NP}}(L)$ was attributed to the thermal vibration energy of atoms at the grain boundary being lower than that at the surface. The model prediction agrees roughly with the experimental results.

**Figure 2:**
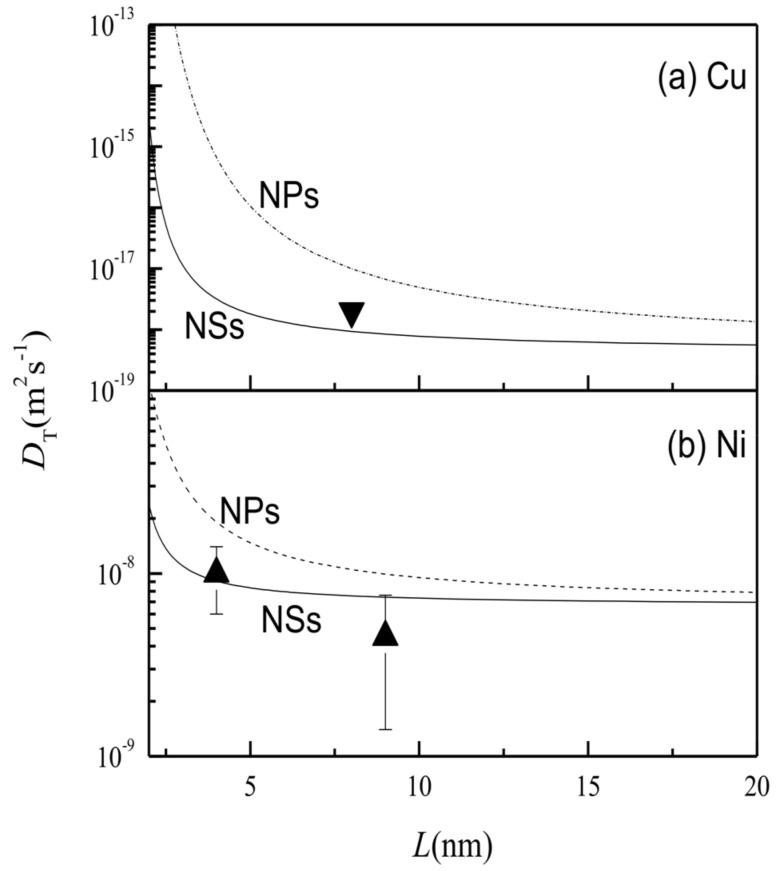
$D_{T}^{\mathrm{NS}}\left(L,T\right)$ as a function of *L* (solid) in terms of Equation (6) for (**a**) Cu and (**b**) Ni; $D_{T}^{\mathrm{NP}}\left(L,T\right)$ functions (dashed) are also plotted with Equations (1) and (2) for comparison. The symbols denote the experimental results with ▼ [27] for Cu NSs and ▲ [16] for Ni NSs. The parameters for the calculation are shown in Table 1.

Based on the results in Figure 1, Figure 2 further depicts the $D_{T}^{\mathrm{NS}}\left(L,T\right)$ functions of (a) Cu and (b) Ni in terms of Equation (6) for NSs in comparison to available experimental results. The case of $D_{T}^{\mathrm{NP}}\left(L,T\right)$ is also plotted with Equations (1) and (2). $D_{T}^{\mathrm{NS}}\left(L,T\right)$ increased on lowering *L* to *L*_0_, where $D_{T}^{\mathrm{NP}}\left(L,T\right)$ > $D_{T}^{\mathrm{NS}}\left(L,T\right)$ > *D_T_* (∞,*T*). An obvious increase of $D_{T}^{\mathrm{NS}}\left(L,T\right)$ occurred at about *L* ≈ 4 nm, although the increase of $D_{T}^{\mathrm{NP}}\left(L,T\right)$ happened at around *L* ≈ 6 nm. When *L* > 10 nm for NSs or *L* > 20 nm for NPs, the values of $D_{T}^{\mathrm{NS}}\left(L,T\right)$ and $D_{T}^{\mathrm{NP}}\left(L,T\right)$ approached *D_T_* (∞, *T*). The increase in $D_{T}^{\mathrm{NS}}\left(L,T\right)$ is related to the decrease of $E_{a}^{\mathrm{NS}}(L)$ because of the coordination imperfection at grain boundaries. It can also be seen that $D_{T}^{\mathrm{NS}}\left(L,T\right)$ < $D_{T}^{\mathrm{NP}}\left(L,T\right)$, which is attributed to the fact that the thermal vibration of atoms at the grain boundary is weaker than at the surface. The model prediction is consistent with the experimental results.

**Figure 3:**
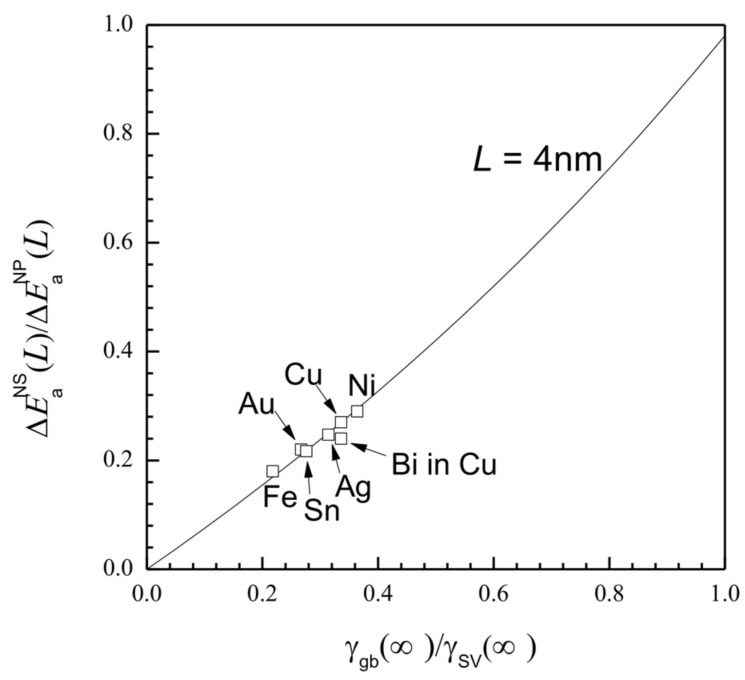
$\Delta E_{a}^{\mathrm{NS}}(L)/\Delta E_{a}^{\mathrm{NP}}(L)$ as the function of *γ*_gb_ (∞)/*γ*_sv_ (∞), with *L* = 4 nm in terms of Equations (4) and (5) for seven elements. The averaged values of *S*_vib_ and *L*_0_ among these seven elements are used for the calculation with *S*_vib_ = 7.251 Jmol^−1·^K^−1^ and *L*_0_ = 1.578 nm. Other parameters are shown in Table 1.

In light of Equations (4) and (5), $E_{a}^{\mathrm{NS}}\left(L\right)$ can be affected by the *γ*_gb_ (∞)/*γ*_sv_ (∞) ratio. To show how $E_{a}^{\mathrm{NS}}\left(L\right)$ will vary with *γ*_gb_ (∞)/*γ*_sv_ (∞), Figure 3 shows a plot of $\Delta E_{a}^{\mathrm{NS}}(L)/\Delta E_{a}^{\mathrm{NP}}(L)$ as the function of *γ*_gb_ (∞)/*γ*_sv_ (∞) at *L* = 4 nm using Equations (4) and (5) with $\Delta E_{a}^{\mathrm{NS}}(L)/\Delta E_{a}^{\mathrm{NP}}(L)$ = $\left[E_{a}^{\mathrm{NS}}(L)-E_{a}\left(\infty \right)]\;/\;[E_{a}^{\mathrm{NP}}(L)-E_{a}\left(\infty \right)\right]$. It can be seen that $\Delta E_{a}^{\mathrm{NS}}(L)/\Delta E_{a}^{\mathrm{NP}}(L)$ increases almost linearly as *γ*_gb_ (∞)/*γ*_sv_ (∞) rises. Since 0 < $\Delta E_{a}^{\mathrm{NS}}(L)/\Delta E_{a}^{\mathrm{NP}}(L)$ < 1 exists in the range 0 < *γ*_gb_ (∞)/*γ*_sv_ (∞) < 1, $\Delta E_{a}^{\mathrm{NS}}(L)$ < $\Delta E_{a}^{\mathrm{NP}}(L)$ is available in the whole *γ*_gb_ (∞)/*γ*_sv_ (∞) range, while $\Delta E_{a}^{\mathrm{NS}}(L)$ approaches $\Delta E_{a}^{\mathrm{NP}}(L)$ as *γ*_gb_ (∞)/*γ*_sv_ (∞) tends to be in unity. Thus, the weakening of the $\Delta E_{a}^{\mathrm{NS}}(L)$ function can be scaled by the *γ*_gb_ (∞) size.

**Figure 4:**
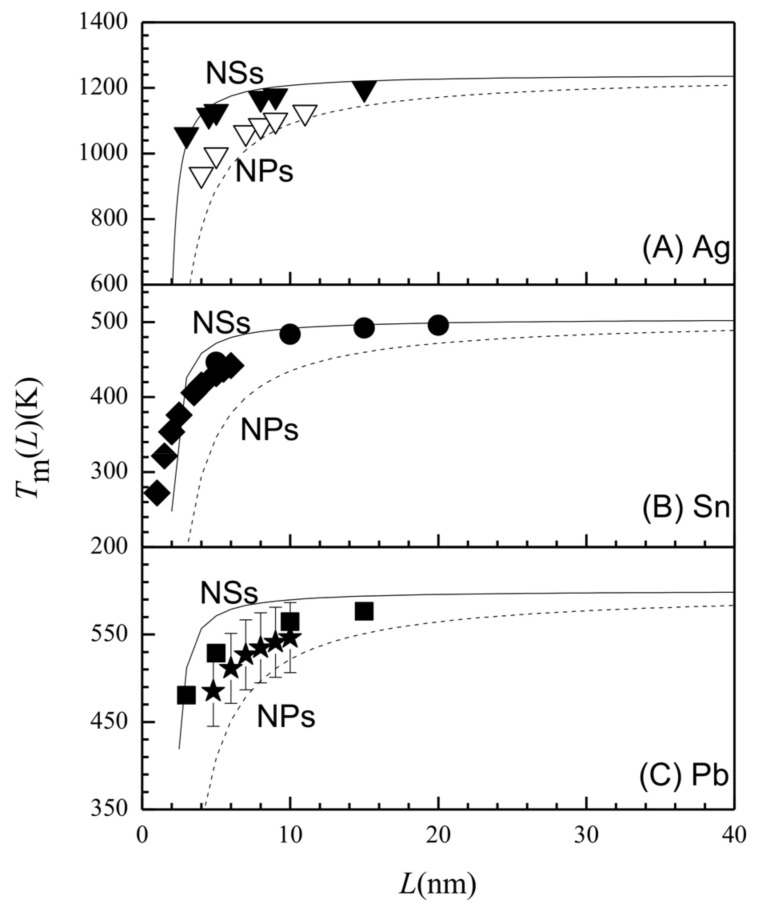
$T_{m}^{\mathrm{NS}}\left(L\right)$ as the function of *L* (solid) in terms of Equation (5) for (**a**) Ag, (**b**) Sn, and (**c**) Pb, where the case of $T_{m}^{\mathrm{NP}}\left(L\right)$ (dashed) is also given for comparison with Equation (2). The symbols show experimental or simulation results with (**a**) ▼ [7] for Ag NSs and ▽ [32] for Ag NPs, (**b**) ◆ [33] and ● [34] for Sn NSs, and (**c**) ■ [35] for Pb NPs and ★ [33] for Pb NSs. The parameters necessary for the calculation are shown in Table 1.

In light of Equation (6), the decrease in $E_{a}^{\mathrm{NS}}\left(L\right)$ and the increase in $D_{T}^{\mathrm{NS}}\left(L,T\right)$ essentially originated from the melting depression of $T_{m}^{\mathrm{NS}}\left(L\right)$, which will be verified here. Figure 4 shows $T_{m}^{\mathrm{NS}}(L)$ functions of (a) Ag, (b) Sn, and (c) Pb in terms of Equation (5) in comparison with the experiment or simulation results. The $T_{m}^{\mathrm{NP}}(L)$ curves are also plotted with Equation (2) for the comparison purpose. $T_{m}^{\mathrm{NS}}\left(L\right)$ decreased as *L* declined with $T_{m}^{\mathrm{NP}}\left(L\right)$ < $T_{m}^{\mathrm{NS}}\left(L\right)$ < *T_m_* (∞), and a significant decrease occurred at about *L* ≈ 5 nm for $T_{m}^{\mathrm{NS}}\left(L\right)$ but at around *L* ≈ 10 nm for $T_{m}^{\mathrm{NP}}\left(L\right)$. The *T_m_* (*L*) value was closer to *T_m_* (∞) when *L* > 10 nm for the NSs or *L* > 20 nm for the NPs. The decrease in *T_m_* (*L*) is related to the coordination imperfection at the interface and the surface. Regarding $T_{m}^{\mathrm{NP}}\left(L\right)$ < $T_{m}^{\mathrm{NS}}\left(L\right)$, this could be due to the fact that *γ*_gb_ (∞) < *γ*_sv_ (∞), since the coordination imperfection at the grain boundary is weak relative to that at the surface. The validity of Equation (5) can be confirmed by the available experiments and computer simulation results, showing that the *L*-dependences of $E_{a}^{\mathrm{NS}}\left(L\right)$ and $D_{T}^{\mathrm{NS}}\left(L,T\right)$ can be influenced by the grain boundary energy effect.

It should be noted, that a degree of deviation can be observed in Figure 1, Figure 2, and Figure 4. Such a deviation can be explained by the error in the grain size measurement. The grain size detected by transmission electron microscopy is larger than that detected by X-ray diffraction [36]. Measurement techniques can also lead to errors in the thermodynamic amounts. In addition, the deviation may also be caused by contributions from the diffusion shortcuts (such as dislocation) and the elastic stress, which should be considered further in future.

## 4. Conclusions

In this paper, we investigated the atomic diffusion coefficient in NSs by considering the role of the grain boundary energy. When *L* was decreased, a decrease in the atomic diffusion activation energy was observed, which led to an increase in the corresponding diffusion coefficient. However, relative to nanoparticles, the variation in the atomic diffusion activation energy of a nanostructured material is small, and is associated with the ratio of the grain boundary energy to the surface energy; the atomic diffusion activation energy of NSs approaches that of nanoparticles, if the grain boundary energy is close to the surface energy. The model prediction for the above functions is in fair accord with the experimental results.

## Figures and Tables

**Table 1:** The necessary data used for the calculations.

	*h* [29] (nm)	*T_m_* (∞) [30] (k)	*S*_vib_ (∞) [29] (Jmol^−1·^k^−1^)	*γ*_sv_ (∞) [24] (Jm^−2^)	*γ*_gb_ (∞) [24] (Jm^−2^)	$D_{T}^{0}(m^{2}\cdot s^{-1})$	*E_a_* (∞) (kJ·mol^−1^)
Ag	0.289	1234	7.82	1.250	0.392	-	-
Pb	0.350	600.61	6.65	0.600	0.111	-	-
Sn	0.281	505.08	9.22	0.649	0.179	-	56.93 [31]
Fe	0.248		6.82	2.420	0.528	-	79.11 [4]
Au	0.288		7.62	1.500	0.400	-	169.81 [22]
Cu	0.256		7.85	1.790	0.601	-	95.52 [15]
						2 × 10^−18^ [27]	66.57 [27]
Ni	0.249		8.11	2.380	0.866	1.77 × 10^−7^ [16]	43.65 [16]
